# Physical Activity, Body Appreciation, and Perceived Stress in Relation to Life Satisfaction Among University Students

**DOI:** 10.3390/healthcare14111572

**Published:** 2026-06-04

**Authors:** Vojko Vučković, Tanja Kajtna, Klemen Širok

**Affiliations:** 1 Faculty of Sport, University of Ljubljana, 1000 Ljubljana, Slovenia; 2 Faculty of Health Sciences, University of Primorska, 6310 Izola, Slovenia; klemen.sirok@fvz.upr.si

**Keywords:** physical activity, life satisfaction, perceived stress, body appreciation, university students, structural equation modelling, well-being

## Abstract

Background: University students experience increased psychological distress during academic transitions, yet modifiable lifestyle determinants of their subjective well-being remain incompletely understood within integrated analytical frameworks. Methods: A cross-sectional survey (N = 194 undergraduates; 52.6% women; M age = 21.9 years) used validated instruments: the Satisfaction with Life Scale (SWLS), Perceived Stress Scale (PSS-10), Body Appreciation Scale-2 Short Form (BAS-2SF), Brief Resilience Scale (BRS), International Physical Activity Questionnaire Short Form (IPAQ-SF), and single-item measures of financial security and screen time. Physical activity (PA) was log-transformed (MET_log). Multiple simultaneous regression and structural equation modelling (SEM) were conducted. Results: Perceived stress was the strongest negative predictor of life satisfaction (B = −0.561, *p* < 0.001), while financial security was a significant positive predictor (B = +0.171, *p* = 0.023). SEM showed that body appreciation was associated with life satisfaction primarily through lower perceived stress (indirect effect = 0.107; consistent with indirect-only association pattern), while PA showed a significant direct association with life satisfaction (β = +0.143, *p* = 0.030), independent of the stress pathway. The indirect effect of PA via stress was not significant in the SEM. Model fit was acceptable (CFI = 0.951; RMSEA = 0.067). Conclusions: Perceived stress was statistically associated with the relationship between body appreciation and subjective well-being, while PA showed a direct statistical association with well-being that was independent of the stress pathway. Given the cross-sectional nature of this study, these findings suggest that university health promotion programmes may consider integrating positive body image and stress management components alongside PA promotion to support student psychological well-being.

## 1. Introduction

The psychological well-being of university students has received sustained research attention over the past two decades, driven by converging evidence that this population bears a disproportionate burden of stress, anxiety, and reduced life satisfaction [1,2]. The transition to higher education is a period of personal reorganisation: academic demands intensify, social networks are rebuilt, financial responsibilities increase, and parental support diminishes. It is therefore unsurprising that psychological distress is consistently more prevalent among undergraduates than in the general young adult population [3]. However, what remains less well understood is the precise configuration of modifiable psychological and lifestyle factors associated with where on the continuum of well-being any given student will fall.

Among lifestyle factors, habitual PA has received the most empirical attention. A substantial body of evidence, including several recent systematic reviews and meta-analyses, confirms that regular moderate-to-vigorous PA is inversely associated with depressive and anxiety symptoms and positively associated with subjective well-being in non-clinical adult populations [4,5]. Neurobiological explanations involve monoaminergic neurotransmission, hypothalamic–pituitary–adrenal axis modulation, and endocannabinoid signalling, while psychosocial explanations emphasise self-efficacy gains, opportunities for social contact, and positive distraction from ruminative thought [6]. In the student context, Pengpid and Peltzer [7] reported that insufficient PA was independently associated with poor self-rated health among university students across 26 low- and middle-income countries. More recent work by Romero-Blanco et al. [8] documented dramatic declines in student PA during the COVID-19 pandemic, alongside corresponding deteriorations in mental health.

Body appreciation, the extent to which individuals hold favourable, accepting attitudes towards their own bodies, [9] has been associated with higher self-esteem and more adaptive health behaviours [10]. Notably, recent research suggests that body appreciation may buffer against psychological distress by reducing the affective impact of stressors, functioning as a cognitive-affective resilience resource [11]. Swami et al. [12] found that higher body appreciation was linked to reduced stress reactivity in everyday life, and Linardon et al. [13], in a 2022 meta-analysis, confirmed that positive body image is strongly associated with greater well-being across diverse samples. However, the mechanisms by which body appreciation confers these benefits, specifically whether stress reduction serves as a significant mediating pathway to life satisfaction, have not been examined within path-analytic models in student samples.

Perceived stress, defined as the extent to which individuals view their circumstances as unpredictable, uncontrollable, and overwhelming [14], is one of the most consistently identified negative predictors of life satisfaction among university students. Cohen and Williamson’s [14] transactional perspective emphasises that it is not the objective stressor load itself, but individuals’ cognitive appraisal of their coping capacity, that determines psychological outcomes. This perspective has direct relevance for interventions, as stress appraisals can be modified through both cognitive–behavioural and lifestyle-based approaches. A recent longitudinal study by Wathelet et al. [15] found that high perceived stress at university entry predicted persistent well-being deficits throughout the first academic year, highlighting the importance of early identification and intervention.

Financial insecurity is an additional, often underappreciated determinant of student psychological well-being. The rising cost of tertiary education, together with student debt, insecure part-time employment, and housing instability, creates chronic financial stress that can overshadow academic engagement and diminish quality of life [3,16]. Financial strain has been linked to higher dropout rates and lower degree attainment [16], but its direct relationship with subjective well-being, after accounting for psychological and lifestyle factors, has not been sufficiently modelled. A 2022 study in *Healthcare* by Aluh and Onu [17] identified financial difficulties as a significant, independent predictor of psychological distress among African university students, and similar patterns have been observed in European samples, though rarely within integrative statistical models that control for behavioural variables.

Taken together, the foregoing bodies of literature converge on a theoretically coherent model that has not, to our knowledge, been explicitly articulated or tested in the student well-being context. The model rests on three interlocking theoretical propositions. First, Cohen’s transactional model of stress [14] holds that psychological distress arises not from objective stressors but from subjective appraisals of a mismatch between demands and coping resources; subjective well-being is therefore mediated by appraisal processes rather than determined directly by environmental conditions. Second, Tylka and Wood-Barcalow’s positive body image framework [10] proposes that body appreciation attenuates appearance-related self-evaluative threat by decoupling self-worth from bodily appearance, thereby reducing the frequency and intensity of stress-amplifying cognitive appraisals. Placed within the transactional model, this generates a specific and testable prediction: body appreciation would be expected to be associated with lower perceived stress, which in turn would be associated with higher life satisfaction, forming a sequential indirect pathway. Third, PA is proposed to exert an independent direct effect on life satisfaction through neurobiological (monoaminergic, endocannabinoid) and psychosocial (self-efficacy, social contact) mechanisms [4,6] that are largely orthogonal to the stress appraisal pathway. This theoretical architecture implies a model with two distinct routes to life satisfaction: an indirect pathway (body appreciation → stress reduction → well-being) and a direct pathway (PA → well-being), with financial security functioning as an additional contextual direct predictor. While regression provides a useful estimate of unique variance, a structural framework allows for the simultaneous estimation of stress as both an outcome and a predictor, facilitating the decomposition of direct and indirect associations.

Several analytical gaps motivate the present investigation. First, while individual associations between PA, body image, stress, and well-being have been documented, their simultaneous, interrelated examination within a structural framework capable of decomposing direct from indirect effects is rare in student samples. Second, the specific hypothesis that body appreciation is associated with life satisfaction through stress reduction has not been formally tested via mediation analysis in this population. Third, financial security, a contextual variable with policy implications, is seldom included alongside psychological and behavioural predictors in integrative well-being models. Fourth, specific mediation hypothesis, that body appreciation is associated with lower perceived stress, which in turn is associated with higher life satisfaction, has not been formally tested as a complete structural model in a student sample. Existing studies either examine body appreciation and stress separately as predictors of well-being [11,13] or test PA and stress in isolation [4,5], but none has simultaneously estimated the direct pathway from PA and the stress-mediated pathway from body appreciation within a single model, nor determined whether their contributions to life satisfaction are statistically independent of one another, the theoretical proposition that body appreciation shapes well-being through a stress reduction mechanism, rather than directly, cannot be adequately tested by regression alone, which estimates all predictors against a single outcome simultaneously. Only a structural framework that models stress as both an outcome (of body appreciation) and a predictor (of life satisfaction) can determine whether the indirect pathway accounts for observed associations and whether PA operates through a separate, direct mechanism.

The present study addressed these gaps by examining these relationships using simultaneous multiple regression and structural equation modelling in a cross-sectional sample of Slovenian university students. The following hypotheses were tested:

**H1.**  *Perceived stress (PSS-10) will be the strongest negative predictor of life satisfaction (SWLS), such that students reporting higher stress appraisals will report significantly lower SWLS scores, after controlling for all other predictors.*

**H2.**  *Physical activity (PA) will demonstrate a significant positive direct effect on life satisfaction (SWLS) in the SEM, independent of the stress pathway.*

**H3.**  *Body appreciation (BAS-2SF) will be associated with life satisfaction (SWLS) indirectly through its negative effect on perceived stress, consistent with a stress-mediation model; its direct effect on SWLS will be non-significant.*

**H4.**  *Financial security will retain a significant positive direct association with life satisfaction (SWLS) after controlling for psychological and behavioural variables.*

## 2. Materials and Methods

### 2.1. Study Design and Participants

A cross-sectional observational study was conducted at two Slovenian universities during the 2025–2026 academic year. Participants were recruited via convenience sampling from students attending the University of Ljubljana and the University of Primorska. Eligibility required full-time enrolment and an age of 20–35 years. Of 220 surveys distributed, 194 were returned with sufficient completeness for analysis (response rate: 88.2%; 52.6% women; mean age = 21.9 years, SD = 2.1). All participants provided written informed consent and the study protocol was approved by the Commission of the University of Primorska for Ethics in Human Subjects Research, number 4264-19-6/23. The procedures conformed to the Declaration of Helsinki.

### 2.2. Measures

Life satisfaction was assessed with the Satisfaction with Life Scale (SWLS) [18], a five-item instrument requiring responses on a seven-point Likert scale (1 = strongly disagree to 7 = strongly agree). The SWLS is among the most widely validated instruments for assessing global cognitive judgements of personal life quality. A validated Slovenian adaptation was used [19]. Internal consistency in the present sample was acceptable (Cronbach’s α = 0.75).

Perceived stress was measured with the ten-item Perceived Stress Scale (PSS-10) [14], which captures appraisals of situations as stressful, uncontrollable, and overwhelming over the past month. A validated Slovenian adaptation was used, and the scale demonstrated adequate reliability in the current sample (α = 0.84).

PA was assessed using the short form of the International Physical Activity Questionnaire (IPAQ-SF) [20], which estimates weekly metabolic equivalent task minutes (MET-min/week) across vigorous, moderate, and walking activity domains. Scoring followed standard IPAQ procedures. The raw MET-min/week distribution exhibited positive skew (skewness = 1.07), a wide range (0 to 19,278 MET-min/week), and 11 zero values; such distributional properties can inflate regression residuals and increase the undue influence of extreme observations. MET-min/week values were therefore log-transformed prior to inferential analyses, a standard procedure for right-skewed PA data [20], which reduced skewness to an acceptable level (skewness = −0.13 after transformation).

Body appreciation was assessed with the Body Appreciation Scale-2 Short Form (BAS-2SF) [9], a validated condensed version of the original BAS-2 [10]. The BAS-2SF has demonstrated strong factorial validity and measurement invariance across gender in international samples. For the purposes of the present study, a Slovenian version was developed following a rigorous forward–backward translation protocol: the scale was first translated into Slovenian by two independent bilingual researchers, reconciled into a single version, then back-translated into English by a third researcher blind to the original. Discrepancies were resolved by consensus. The resulting Slovenian BAS-2SF demonstrated good internal consistency in the present sample (α = 0.86).

Resilience was measured using the validated Slovenian version of the Brief Resilience Scale (BRS) [21], a five-item instrument (five-point response scale) assessing the ability to recover from stress and adversity. The BRS demonstrated adequate reliability in the current sample (α = 0.76).

Financial security was measured with a single item asking participants to rate their overall financial situation on a five-point scale from 1 (very insecure) to 5 (very secure). Single-item financial self-assessment measures have demonstrated acceptable convergent validity with multi-item financial well-being instruments [16].

Screen time was self-reported as the average daily hours of recreational screen use, explicitly excluding academic computing activities.

### 2.3. Statistical Analysis

Descriptive statistics (means, standard deviations) were computed for all study variables. Internal consistency of multi-item scales was evaluated using Cronbach’s alpha, with values ≥ 0.70 considered acceptable [22]. Raw PA values (MET-min/week) were positively skewed and were therefore log-transformed prior to all inferential analyses.

Multiple linear regression was used to identify independent predictors of SWLS. All seven predictors (PSS-10, BAS-2SF, PA, BRS, financial security, gender, and screen time) were entered simultaneously in a single block to obtain adjusted regression coefficients. Multicollinearity was assessed using variance inflation factors (VIFs); all VIF values were below 3.0, indicating no problematic collinearity.

Structural equation modelling (SEM) was subsequently conducted using the semopy package in Python (version 2.3.11 [23]) to simultaneously estimate direct and indirect pathways among PA, body appreciation, perceived stress, financial security, and life satisfaction. Model fit was evaluated using the Comparative Fit Index (CFI; ≥0.95 = good, ≥0.90 = acceptable), Tucker–Lewis Index (TLI; ≥0.90 = acceptable), Root Mean Square Error of Approximation (RMSEA; ≤0.08 = acceptable), and chi-square probability value (*p* > 0.05 = acceptable fit). Indirect effects were estimated using bias-corrected bootstrap confidence intervals (5000 resamples) [24,25,26]. The significance threshold was set at α = 0.05 for all analyses [23]. Maximum likelihood estimation was used. The assumption of multivariate normality was evaluated by inspecting skewness and kurtosis of all endogenous variables, which fell within acceptable limits (|skewness| < 2, |kurtosis| < 7). Observations were independent by design. The model was intentionally parsimonious and specified to test four a priori hypotheses; moderated mediation and additional mediators represent natural extensions for future research with larger samples. Post hoc power considerations are addressed in the Limitations section.

## 3. Results

### 3.1. Descriptive Statistics and Internal Consistency

Table 1 presents the descriptive statistics and reliability estimates for all study measures. Life satisfaction scores (SWLS) showed a mean of 4.64 (SD = 1.07), broadly consistent with values reported in other Central and Eastern European student samples [27]. Perceived stress was moderately elevated (PSS-10: M = 2.74, SD = 0.56), while body appreciation was near the scale midpoint (BAS-2SF: M = 3.71, SD = 0.94). PA exhibited considerable variability in raw MET-min/week (M = 5368.24, SD = 3424.99). Log-transformed values (MET_log) were used in all analyses, except in descriptive statistics (Table 1). All multi-item scales exceeded the α = 0.70 threshold, confirming adequate-to-excellent internal consistency.

### 3.2. Multiple Regression Analysis

The simultaneous multiple regression model was statistically significant, F(7, 186) = 7.07, *p* < 0.001, accounting for 21.0% of the variance in life satisfaction (R^2^ = 0.210; adjusted R^2^ = 0.180). Table 2 presents the unstandardised and standardised regression coefficients for each predictor.

Perceived stress was the strongest individual predictor (B = −0.561, β = −0.297, *p* < 0.001). Financial security showed a significant positive association (B = 0.171, β = 0.166, *p* = 0.023). Gender was also significant (B = 0.315, β = 0.148, *p* = 0.045), female students scoring higher in life satisfaction. PA showed a significant positive association with life satisfaction (B = 0.073, β = 0.140, *p* = 0.036). The remaining predictors—body appreciation, resilience, and screen time—did not reach significance when all variables were held constant. The negative BRS coefficient reflects a statistical suppression effect attributable to its strong negative correlation with PSS-10 (r = −0.54); once perceived stress was controlled, resilience contributed no unique predictive variance (zero-order r = 0.117, *p* = 0.103).

### 3.3. Structural Equation Model

SEM was employed to model two simultaneous equations, one predicting perceived stress (PSS-10) and the second predicting life satisfaction (SWLS), enabling the decomposition of direct and indirect pathways. Table 3 presents the standardised path coefficients.

Although resilience was examined as a predictor of life satisfaction in the preliminary regression analyses, it was excluded from the SEM on the basis of its non-significant contribution (β = −0.068, *p* = 0.390) and to preserve model parsimony given the available sample size.

In the stress equation, body appreciation was the largest standardised coefficient (β = −0.503, *p* < 0.001). Higher BAS-2SF scores were associated with substantially lower perceived stress. PA showed a trend but did not significantly predict PSS-10 once BAS-2SF was controlled (β = −0.064, *p* = 0.302).

In the life satisfaction equation, perceived stress showed a significant negative direct association (β = −0.212, *p* = 0.005). PA demonstrated a significant positive direct association with SWLS (β = 0.143, *p* = 0.030), independent of the stress pathway. Financial security retained its direct positive association with SWLS (β = 0.188, *p* = 0.007). Body appreciation showed no significant direct effect on SWLS (β = 0.087, *p* = 0.274).

Formal model comparison supported the constrained specification (direct path BAS-2SF → SWLS fixed to zero): the constrained and unconstrained models did not differ significantly (Δχ^2^(1) = 1.26, *p* = 0.262), and both AIC and BIC favoured the constrained model. The bootstrapped 95% CI for the direct effect included zero (β = 0.087, 95% CI [−0.083, 0.251]), consistent with an indirect-only association pattern [26]. However, given N = 194, the study was likely underpowered to detect a small direct effect, and this null result should be interpreted with caution [24,25]. Path decomposition revealed an indirect-only mediation pattern: the indirect effect of body appreciation on life satisfaction via perceived stress was significant (indirect = 0.107, 95% CI [0.026, 0.199]), while the direct effect was non-significant (β = 0.087, 95% CI [−0.083, 0.251]); total effect = 0.194.

Overall model fit was acceptable: CFI = 0.951, TLI = 0.910, RMSEA = 0.067, χ^2^(*p*) = 0.069. The chi-square *p*-value exceeded 0.05, meaning that the hypothesis of exact fit could not be rejected.

## 4. Discussion

The present study aimed to examine the interrelated contributions of PA, body appreciation, perceived stress, resilience, and financial security to life satisfaction in a sample of Slovenian undergraduate students, using an analytical strategy capable of distinguishing direct and mediated effects. Overall, the findings present a theoretically coherent associational picture: perceived stress showed the strongest statistical association with body appreciation’s relationship to well-being, while PA and financial security were each independently associated with life satisfaction. Each of the four hypotheses received empirical support, though with important qualifications that require careful interpretation given the cross-sectional design.

### 4.1. Perceived Stress and Life Satisfaction

The primacy of perceived stress as a negative predictor of life satisfaction (H1 supported: B = −0.561 in regression; β = −0.212 as a path in SEM) is entirely consistent with decades of research in the stress and well-being literature [14,27]. It is worth considering why stress appraisal, rather than objective stressor count, carries such explanatory weight. Cohen and Williamson’s transactional model highlights that two students facing the same examination schedule may differ greatly in their appraisal of whether they have sufficient resources to cope, and it is this gap between perceived demands and perceived resources that drives distress [14]. This has immediate practical consequences: interventions do not necessarily need to reduce the objective stressor environment (often impossible within institutional structures) but may achieve meaningful gains by enhancing students’ appraisal of their own coping capacity. A large-scale survey by Larcombe et al. [28] identified high levels of perceived stress among university students. The authors concluded that students entering higher education are at increased risk and that psychological stress may undermine academic functioning. The present findings are consistent with this concern.

### 4.2. Physical Activity’s Direct Pathway

The significant direct association of PA with life satisfaction in the SEM (H2 supported: β = +0.143, *p* = 0.030) is noteworthy, though the cross-sectional design precludes causal inference. Notably, in the SEM, PA did not show a significant indirect association via stress reduction (indirect = 0.014, 95% CI [−0.019, 0.059]), suggesting that its association with well-being may operate through pathways other than stress reduction. Standard neurobiological accounts of the exercise–mood relationship invoke monoaminergic neurotransmission and endocannabinoid signalling [6]; however, these were not measured in the present study and cannot be inferred from the observed path coefficient. They remain theoretically plausible explanations requiring experimental confirmation. PA reached significance in both regression (B = 0.073, *p* = 0.036) and SEM (β = 0.143, *p* = 0.030), providing evidence of a direct positive association with life satisfaction across both approaches. A 2023 study in *Healthcare* by Romero-Blanco et al. [8] similarly found that PA was a significant predictor of well-being after controlling for stress in a Spanish student sample, lending cross-cultural support to the present finding.

### 4.3. Body Appreciation as a Stress Buffer

The indirect-only association pattern for body appreciation’s relation to life satisfaction via perceived stress (H3 supported: indirect association = 0.107, direct association = 0.087, ns) represents the study’s most theoretically informative finding. The total effect of BAS-2SF on SWLS was not negligible (total = 0.194) but was expressed almost entirely through the reduction in perceived stress. This finding is consistent with positive body image theory [10], which posits that appreciating one’s body confers psychological resilience because it disrupts appearance-related rumination and self-critical appraisals that amplify the subjective experience of stress. Students who hold favourable, non-contingent attitudes towards their bodies may be less likely to interpret challenging daily events—a poor examination result, a social rebuff, or financial difficulty—through the lens of bodily inadequacy, thereby buffering the stress appraisal process. Linardon et al.’s [13] meta-analysis found that positive body image was associated with lower internalised thin-ideal and appearance comparison, both of which are known stress amplifiers, a mechanism that aligns well with the path observed in this study. To our knowledge, this specific cross-sectional association pattern—BAS → PSS → SWLS—has not previously been reported in a Slovenian student sample, and it invites replication in other cultural contexts.

### 4.4. Financial Security

Financial security’s independent direct contribution to life satisfaction (H4 supported: β = 0.188, *p* = 0.007 in SEM) remained significant after adjustment for all psychological and behavioural covariates, indicating that its benefits are not fully accounted for by the stress pathway. This pattern is consistent with direct hedonic mechanisms. Financial stability may provide greater autonomy in daily decision-making, reduce exposure to chronic uncertainty, and enable access to health-promoting goods and social activities, although such mechanisms cannot be confirmed from cross-sectional data. Aluh and Onu’s [17] 2022 study identified financial difficulties as a significant predictor of psychological distress, and the present data extend this pattern by showing that the inverse association between financial security and life satisfaction persisted even when perceived stress was modelled concurrently. These findings suggest that financial welfare structures may be worth exploring as potential contributors to student psychological well-being, alongside psychological support, although causal claims require longitudinal evidence.

The absence of a significant direct association for resilience (BRS) in either analytic approach warrants comment. The zero-order correlation between BRS and SWLS was positive and non-significant (r = 0.117, *p* = 0.103, 95% CI [−0.024, 0.254]), yet the regression coefficient was negative (B = −0.135), a pattern diagnostic of suppression. BRS and PSS-4 are substantially negatively correlated (r = −0.54), and once stress was controlled, BRS contributed no unique variance to SWLS prediction. This does not imply that resilience is unimportant for student well-being. A substantial body of evidence clearly supports resilience as a meaningful psychological resource, particularly in the face of acute adversity [2,21]. Rather, it suggests that in the present model, resilience operates through its association with perceived stress rather than as an independent predictor of life satisfaction. Future research may warrant consideration to examine resilience as a moderator of the stress–well-being pathway rather than as a direct predictor.

#### Limitations

Several limitations constrain interpretation. First, the cross-sectional design precludes causal inference; although the directional hypotheses are theory-based, bidirectional and reverse-causal explanations remain plausible [4,29]. Students who are more satisfied with life may exercise more, appreciate their bodies more, and experience less stress. Additionally, the relationship between life satisfaction and perceived stress may be bidirectional: students with higher well-being may appraise the same stressors as less threatening, and body appreciation may itself be partially determined by life satisfaction rather than the reverse. The present cross-sectional data cannot distinguish among these competing structures, and the proposed model should be understood as a theoretically motivated starting point for longitudinal investigation rather than a confirmed account of the underlying process. Second, PA was assessed using the IPAQ-SF self-report, which is subject to recall and social desirability bias; future studies would benefit from accelerometry. Third, the sample was drawn from two Slovenian universities using convenience sampling, limiting generalisability to other cultural or national contexts. Both institutions draw from a relatively homogeneous socioeconomic and cultural context, and replication in more diverse samples, including vocational, part-time, and mature-age student populations, is needed before broader conclusions can be drawn. Fourth, the model explained 21.0% of the variance in life satisfaction—a respectable but not comprehensive proportion. Unmodelled variables such as social support quality, sleep duration, academic self-efficacy, and purpose in life likely account for substantial additional variance. Fifth, gender, which emerged as a significant predictor in the regression model (B = 0.315, *p* = 0.045), was not included in the path analysis model. While this decision was driven by the a priori theoretical scope of the study, it is possible that omitting gender introduced modest bias in the estimated path coefficients; future studies may consider examining whether gender moderates any of the hypothesised pathways. Additionally, the study was likely underpowered to detect the small direct path from body appreciation to life satisfaction (β = 0.087), and the null direct association should be interpreted as consistent with indirect-only mediation rather than as proof that the true direct association is zero. The wide confidence interval [−0.083, 0.251] reflects limited precision, and a larger sample would be needed to determine with confidence whether a small direct association exists. Non-significant paths, particularly BAS-2SF → SWLS (β = 0.087), should be interpreted with caution regarding Type II error. Some limitations should be noted regarding the measures used. Financial security was assessed with a single item, limiting its reliability and construct coverage compared to validated multi-item instruments. Although the Slovenian BAS-2SF was developed using a forward–backward translation protocol, no confirmatory factor analysis or test–retest reliability study was conducted. PA was assessed via the IPAQ-SF self-report, which is susceptible to recall and social desirability bias; objective accelerometry-based measurement would be preferable in future studies. Variables with established relevance to student well-being, including social support, sleep quality, academic self-efficacy, and sense of purpose, were not measured and may confound the observed associations. Body appreciation and perceived stress are likely co-determined by unmeasured personality characteristics.

### 4.5. Implications

Despite these limitations, the findings offer hypothesis-generating observations that may inform future research directions for university health promotion. These observations are offered strictly as cross-sectional associational findings and should not be interpreted as evidence-based practice recommendations; replication in longitudinal or experimental designs is needed before practice implications can be drawn. The finding that body appreciation and PA were each associated with well-being through statistically distinct pathways suggests that programmes targeting only one construct may not fully address the range of relevant mechanisms, though this hypothesis requires prospective testing. Integrated approaches exploring both positive body image and PA participation could be examined in future intervention research. Stress management support, whether through cognitive–behavioural therapy, mindfulness-based stress reduction, or peer support, may be considered as a component of university mental health services, given the strong negative association of perceived stress with life satisfaction observed here. Financial welfare structures could be explored as potential contributors to student psychological well-being, a hypothesis that longitudinal research may examine.

## 5. Conclusions

The present study identified perceived stress, body appreciation, PA, and financial security as cross-sectional correlates of life satisfaction in a sample of Slovenian undergraduate students. Specifically, body appreciation was associated with life satisfaction indirectly through lower perceived stress, whereas PA and financial security showed independent direct associations. These findings should be interpreted strictly as associations; the cross-sectional design does not permit causal inference, and the direction and underlying mechanisms of the observed pathways require longitudinal or experimental investigation before they can be established.

## Figures and Tables

**Table 1:** Descriptive statistics and internal consistency of study measures (N = 194).

Variable	M	SD	Skewness	Kurtosis	N Items	α
SWLS	4.64	1.07	−0.68	0.28	5	0.75
PSS-10	2.74	0.56	0.26	−0.40	10	0.84
BAS-2SF	3.71	0.94	−0.51	−0.24	3	0.86
BRS	3.4	0.63	−0.06	−0.30	5	0.76
PA	5368.24	3424.99	1.07	1.82	N/A	N/A
Financial Security	2.9	1.1	−0.20	−0.47	N/A	N/A
Screen Time (h/day)	4.3	2.1	−0.01	0.43	N/A	N/A

Note. SWLS = Satisfaction with Life Scale; PSS-10 = Perceived Stress Scale-10; BAS-2SF = Body Appreciation Scale-2 Short Form; BRS = Brief Resilience Scale; PA = MET-min/week; N/A = not applicable (single-item or composite variable with no α).

**Table 2:** Multiple regression predicting Satisfaction with Life Scale (SWLS; N = 194).

Predictor	B	SE	β	t	95% CI [B]	*p*
PSS-10	−0.561	0.169	−0.297	−3.31	[−0.90, −0.23]	0.001 ***
Financial Security	0.171	0.075	0.166	2.29	[0.02, 0.32]	0.023 *
Gender (women = 1)	0.315	0.156	0.148	2.02	[0.01, 0.62]	0.045 *
PA (MET_log)	0.073	0.034	0.140	2.11	[0.005, 0.140]	0.036 *
BAS-2SF	0.123	0.091	0.108	1.35	[−0.06, 0.30]	0.179
BRS	−0.116	0.135	−0.068	−0.86	[−0.38, 0.15]	0.390
Screen Time (h/day)	−0.050	0.085	−0.041	−0.593	[−0.217, 0.117]	0.554

Note. R^2^ = 0.210; adjusted R^2^ = 0.180; F(7, 186) = 7.07, *p* < 0.001. Confidence intervals are 95%. * *p* < 0.05; *** *p* < 0.001.

**Table 3:** SEM path coefficients for the two-equation structural model (N = 194).

Path	β	SE	*p*	Boot 95% CI	Interpretation
(1): Outcome = PSS-10					
BAS-2SF → PSS-10	−0.503	0.062	<0.001	[−0.62, −0.38]	Body appreciation is strongly associated with stress
PA (MET_log) → PSS-10	−0.064	0.062	0.302	[−0.23, 0.09]	Non-significant when BAS-2SF is controlled
(2): Outcome = SWLS					
PSS-10 → SWLS	−0.212	0.076	0.005	[−0.38, −0.05]	Higher stress → lower life satisfaction
PA (MET_log) → SWLS	+0.143	0.066	0.030	[0.00, 0.30]	Direct PA association with life satisfaction
FinSec → SWLS	+0.188	0.069	0.007	[0.07, 0.31]	Better finances → higher SWLS
BAS-2SF → SWLS (direct)	+0.087	0.080	0.274	[−0.08, 0.25]	No significant direct association; indirect-only mediation via PSS-10
BAS-2SF → PSS-10 → SWLS (indirect)	+0.107	0.044	<0.001	[0.026, 0.199]	Indirect-only association pattern supported

Note. β = standardised path coefficient. Boot 95% CI = bias-corrected bootstrapped confidence intervals (k = 5000). BAS-2SF = Body Appreciation Scale-2 Short Form; PSS-10 = Perceived Stress Scale-10; SWLS = Satisfaction with Life Scale; PA = physical activity; MET_log = log-transformed MET-min/week; SE = standard error; FinSec = financial security.

## Data Availability

The data presented in this study are openly available in https://github.com/vuckovicvojko/Article, accessed on 30 March 2026.

## Abbreviations

BAS-2SF	Body Appreciation Scale-2 Short Form
BRS	Brief Resilience Scale
CFI	Comparative Fit Index
CI	Confidence Interval
FinSec	Financial Security (Table-Only Abbreviation)
IPAQ-SF	International Physical Activity Questionnaire—Short Form
MET	Metabolic Equivalent of Task
MET_log	Log-Transformed MET-Min/Week
PA	Physical Activity
PSS-10	Perceived Stress Scale-10
RMSEA	Root Mean Square Error of Approximation
SD	Standard Deviation
SEM	Structural Equation Modelling
SWLS	Satisfaction with Life Scale
TLI	Tucker–Lewis Index
VIF	Variance Inflation Factor
